# The bee bread of honey bees is characterized by a core microbiota despite the application of miticide treatments and variation across space and time

**DOI:** 10.7717/peerj.20366

**Published:** 2025-11-20

**Authors:** Brooke L. Lawrence, Gordon F. Custer, Robyn M. Underwood, Robert R. Dunn, Francisco Dini-Andreote, Margarita M. López-Uribe

**Affiliations:** 1 Department of Entomology, Pennsylvania State University, University Park, PA, United States; 2 Department of Plant Science, Pennsylvania State University, University Park, PA, United States; 3 The One Health Microbiome Center, Huck Institutes of the Life Sciences, Pennsylvania State University, University Park, PA, United States; 4 Department of Applied Ecology, North Carolina State University, Raleigh, NC, United States

**Keywords:** *Apis mellifera*, Bacterial community, Microbiome, Formic acid, Amitraz

## Abstract

**Background:**

Bee bread is composed of a mixture of pollen and nectar used as the main source of proteins and lipids for the development of bee larvae. Despite its important role in honey bee food preservation, relatively little is known about the composition of bee bread microbiota and the potential impact of beekeeping management of hives on these microbial systems.

**Methods:**

Here, we evaluated whether (1) the bee bread of honey bees is characterized by a core microbiota and (2) miticide applications (formic acid and amitraz) affect the diversity and composition of the bee bread microbiota. We collected a total of 36 samples from six sites across two distinct geographic locations and sequenced the bee bread bacterial communities before and after miticide applications.

**Results:**

Our results revealed a conserved bee bread core microbiota comprised of 15 taxa belonging to the phyla Proteobacteria (11 taxa), Firmicutes (two taxa), Actinobacteriota (one taxon), and Bacteroidota (one taxon). In addition, we found weak evidence of miticide treatments impacting the diversity of the bee bread microbiota, with a general trend of a decrease in the diversity of non-core taxa following the application of organic miticides.

**Conclusion:**

Taken together, our results demonstrate that the bee bread of honey bees is characterized by a core microbiota despite variations associated with geographic location, sampling period, and miticide applications.

## Introduction

The western honey bee (*Apis mellifera* L.) is a perennial social insect species that stores food in hives to survive long periods of time, particularly when environmental conditions are not optimal for foraging. Honey bee colonies store two different types of food: honey (the main source of carbohydrates) and bee bread (the main source of protein, lipids, vitamins, and minerals) (Haydak, 1970). Honey is a sugar-dense food source composed of processed floral nectar. Worker bees introduce digestive enzymes to the collected nectar to break down the sugars into more easily digestible forms (*e.g*., fructose, glucose, sucrose) and then dehydrate it to further aid its preservation (Brodschneider & Crailsheim, 2010). Honey is a food resource consumed by all worker bees in the colony year-round. Conversely, bee bread is composed of a mixture of pollen, nectar, and saliva and is stored in the hive for nurse bees’ consumption. This protein-rich food is necessary for the development of the nurse bees’ hypopharyngeal glands, which produce a nutrient-rich jelly fed to the developing larvae (de Groot, 1953). In addition, developing larvae are fed bee bread starting on day 4 or 5 of development as a dietary supplement to worker jelly (Haydak, 1970). As such, high-quality (unspoiled) bee bread is essential to the maintenance of individual and overall colony health.

Bee bread contains a microbiota associated with the production of metabolites with antimicrobial properties (*e.g*., lactic and acetic acid) that can help with food preservation and reduction of pathogenic microbes (Anderson et al., 2011). In this way, bee bread is akin to sourdough bread starters in which the acids produced by lactic acid and acetic acid bacteria inhibit the growth of some taxa (*e.g*., Landis et al., 2021). The introduction of environmental microbes to the bee bread microbiota occurs primarily through the dispersal of pollen- and nectar-associated microbes, and from honey bee crops (Anderson et al., 2013; Corby-Harris, Maes & Anderson, 2014). For instance, bacterial species associated with pollen are known to be highly plant species-specific. A study comparing the bacterial diversity of pollen from four phylogenetically distant plant species (*Brassica*, *Secale*, *Betula*, *Colchicum*) found between 130 and 530 bacterial operational taxonomic units (OTUs) with little overlap in the bacterial composition of these different types of pollen (Ambika Manirajan et al., 2016). Thus, the bee bread bacteria that disperse from pollen may largely be influenced by the local abundance of plant species in bloom, which varies temporally and spatially. In contrast, the microbiota of honey bee crops is structured by a sugar-rich acidic environment that acts as a stringent abiotic filter, resulting in a microbiota dominated by two extremophilic bacteria: *Apilactobacillus kunkeei* and *Bombella apis* (formerly known as *Lactobacillus kunkeei* and *Parasaccharibacter apium*, respectively (Corby-Harris, Maes & Anderson, 2014).

Given the important function of the bee bread microbiota in the preservation of stored food, it is likely that a consistent set of species (*i.e*., core taxa) is required to maintain key functions that prevent pollen spoilage. For example, *A. kunkeei* and *B. apis* have been consistently reported in bee bread, and these taxa are known to aid with sugar fermentation (Anderson et al., 2014). The breakdown of sugars in bee bread creates a preservative environment with low pH and decreased oxygen availability, preventing the overgrowth of microbes that can spoil its nutritional quality (Anderson et al., 2013). Because the crop microbiota is highly conserved and composed of a few related lineages that are adapted to the acidic conditions (Olofsson & Vásquez, 2008; Moran, 2015), the addition of these bacteria to the bee bread might confer stability to its functions and may act as a route of repeated dispersal (Disayathanoowat et al., 2020). However, relatively little is known about the existence and composition of a core bee bread microbiota, as well as the degree to which human management of hives impacts these systems (but see Donkersley et al., 2018).

As important pollinators in modern agricultural systems, honey bees and associated microbiota are frequently exposed to pesticides, with the potential for dysbiosis in colonies and food stores (Kakumanu et al., 2016). Indeed, several studies have shown that pollen and bee bread can contain high levels of pesticide residues (Mullin et al., 2010; Calatayud-Vernich et al., 2018, 2019; Roth et al., 2020). However, the effects of these residues on the bee bread microbiota of honey bees in hive environments have not yet been well investigated (Vásquez & Olofsson, 2009; Yoder et al., 2013). Additionally, beekeepers often directly apply miticides to the colony to control populations of Varroa mite—a deadly ectoparasitic pest (Roth et al., 2020). A recent study demonstrated that the use of the fungicide chlorothalonil and the miticides coumaphos and tau-fluvalinate significantly altered the structure and functional potential of the gut microbiota of honey bees (Kakumanu et al., 2016). However, the extent to which other beekeepers’ commonly applied chemical miticides (*e.g*., amitraz and formic acid)—used to control Varroa mites—impact the microbiome of the honey bees and hive food storage remains still to be properly investigated.

In this study, we sought to answer the following questions: (1) Is there a core microbiota associated with the bee bread of honey bees? (2) Does the diversity and composition of the bee bread microbiota vary due to the management of honey bee colonies with miticide treatments? To answer these questions, we established 36 honey bee colonies across six sites in two distinct geographic locations, and collected bee bread samples before and after the application of organic and synthetic miticides (*e.g*., formic acid and amitraz, respectively). We hypothesized that: (1) despite variation in the location of the colonies and the timing of sampling, bee bread has a conserved set of core bacterial taxa; and (2) miticide treatments significantly alter the bacterial community of bee bread in a miticide-dependent manner. Specifically, we expected that the application of organic miticides would increase the abundance of acidophilic taxa due to a reduction in the bee bread pH. Our results support the presence of core bacteria species in bee bread despite the application of miticites, and samples collected at different times and sites. Moreover, and contrary to our prediction, we found the bacterial communities of the bee bread to be generally robust to miticide treatments.

## Materials and Methods

### Experimental design

**Figure 1:**
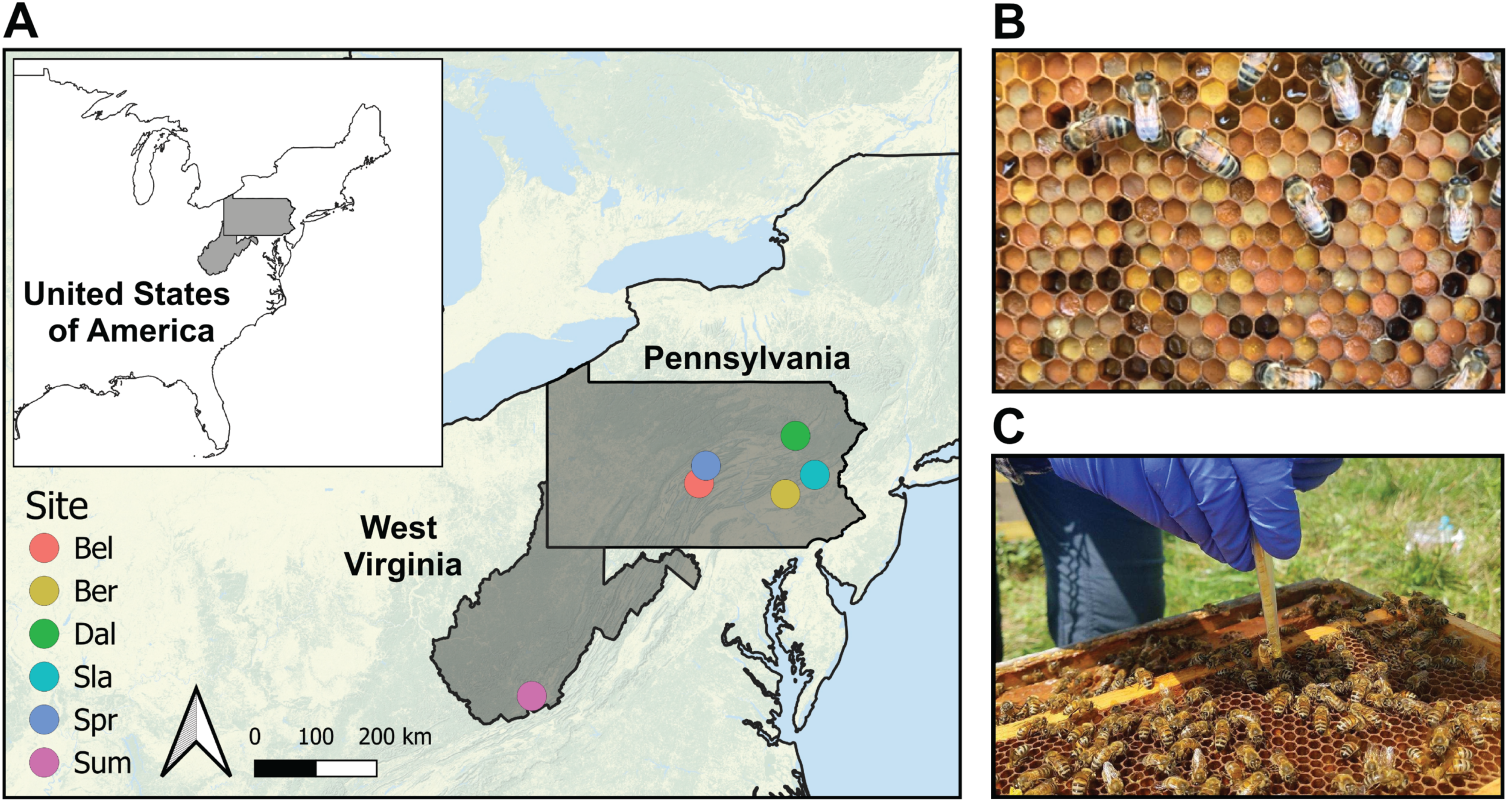
Sampling protocol. (A) Map of the six certified organic farm sites in the states of Pennsylvania and West Virginia (USA). We sampled two colonies per treatment in each of the selected farms for a total sample size of 36 colonies. Details of the geographic coordinates are in Table S1. (B) Bee bread stored in honeycomb. Note differences in pollen color across samples. (C) Procedure for bee bread sampling of 12 cells using a precut pipette tip inserted into the comb cell.

We studied a total of 36 newly managed honey bee colonies located at five farms in Pennsylvania and one in West Virginia (USA) (hereafter referred to as sites) (Fig. 1; Table S1). These areas are dominated by temperate deciduous forests (60–70%), mixed with land used for agriculture and grazing (20–30%), and some low-intensity development. Each colony was established with 1.36 kg (3 lbs) of bees and a mated queen in April 2018 placed in new 10-frame Langstroth equipment with wax-coated plastic foundation frames (detailed methods reported in Underwood et al., 2023). Packages were purchased from two producers in Georgia, USA in April 2018 and requeened in July 2018 with sister queens reared and openly mated near Utica, NY (USA). Colonies were managed using one of the following three management systems: miticide-free (MF; no miticides), conventional (CON; use of a synthetic miticide and organic acids), or organic (ORG; use of organic acids) (Table S2). Each management system was established in two colonies located on the six farms (sites), and samples were collected before and after the miticide application of amitraz in colonies under conventional management, and formic acid in colonies under organic management. Colonies in the miticide-free management were also sampled at the same time, despite the lack of application of miticides, and were used as controls. We selected certified organic farms as the sites for this study to minimize pesticide exposure from outside the colonies.

#### Application of in-hive chemical mite treatments

Three days after package installation, colonies in the organic and conventional management systems were treated with 2.5% oxalic acid in 1:1 sucrose syrup at a rate of 5 ml per frame of bees to a maximum of 50 ml. Thereafter, no chemical treatments were applied until the fall. Fall miticide treatments were applied in late August, as colonies were broken down to their overwintering size (*i.e*., three medium boxes). Colonies in the miticide-free group did not receive any chemical treatment at any time (Table S2). Colonies managed under the conventional system were treated with amitraz following label recommendations. Specifically, four amitraz strips were hung between brood frames in the bottom two boxes leaving strips inside the colony for 42 days (Veto-pharma). Colonies managed under the organic system were treated with formic acid polysaccharide gel strips containing 42.25% formic acid. Two strips were added to each hive on top of the frames for 20 days—one strip added on day 0 and a second strip on day 10—on days when the ambient temperature was between 10 °C and 33.3 °C to avoid excessive colony damage (NOD Apiary Products). In addition to formic acid application, the organic management system included a mite-trapping technique called drone brood removal, where pupal drones, along with the mites reproducing in their cells, were scraped from specialized brood frames every two weeks. These drone frames were removed on the day of formic acid application and replaced with standard, drawn frames.

### Sample collection

Bee bread samples were collected from each colony before (August 2018) and after (Sep 2018) miticide applications—referred to hereafter as pre- and post-treatments. The pre-treatment samples were collected before the miticide treatment was applied (day 0) and the post-treatment samples were collected 20 days after (day 20). The bee bread samples were collected using 12 cells from the outer edges of 3 brood frames per hive using a 1 mL precut pipette tip inserted into the comb cell (Fig. 1C). This sampling method resulted in one pooled sample per colony (*n* = 36 samples per timepoint). Samples were stored in individual sterile 15 mL conical tubes and transported to the laboratory (<24 h) on dry ice and stored at −80 °C.

### DNA extraction

Bee bread samples were removed from pipette tips using a flame-sterilized metal rod and metal weighing spatula. A total of 0.05 g ± 0.008 g of bee bread was transferred to a ZR BashingBead Lysis Tube (Catalog number S6012-50; Zymo Research, Irvine, CA, USA). Total DNA was extracted using the Zymo Quick-DNA Fecal/Soil Microbe Microprep Kit (Catalog number D6012; Zymo Research, Irvine, CA, USA), following the manufacturer’s standard non-soil samples protocol. DNA was eluted in 20 µL of sterile water and stored at −80 °C until the preparation of amplicon libraries. The V4 region of the bacterial 16S rRNA gene was amplified using the primer set 515-F (GTGCCAGCMGCCGCGGTAA) (Walters et al., 2016; Parada, Needham & Fuhrman, 2016) and 806-R (GGACTACHVGGGTWTCTAAT) (Apprill et al., 2015). The PCR contained 6.25 µL of PCR master mix with Taq polymerase, 0.5 µL of 515F primer, 0.5 µL of 806R primer, 4.75 µL MilliQ water, and 1.00 µL sample DNA, with a final volume of 12.5 µL. PCR cycling conditions were 94 °C for 5 min followed by 35 cycles of 94 °C for 45 s, 50 °C for 60 s, and 72 °C for 90 s, and a final extension of 72 °C for 10 min. PCR products were visualized on a 1% agarose gel, stained with SYBR Green Nucleic Acid Stain. Equimolar libraries were sequenced on Illumina’s MiSeq platform using V3 chemistry (2 × 250 PE) at the University of Colorado Next Generation Sequencing Facility (Boulder, Colorado).

### Sequencing data processing

Amplicon sequence data were demultiplexed using the idemp package. Next, primers were removed using Cutadapt (v. 3.7) (Martin, 2011). Sequences were further processed in R using the DADA2 pipeline (Callahan et al., 2016). Trimmed reads were processed using DADA2 for quality filtering, error learning, dereplication, merging, and chimera removal. Briefly, the following quality filtering parameters were used: trunQ = 11, maxEE = c(1,1), and truncLen = c(150,140). All base pairs were used to learn errors (nbases =1 × 10^9^), and sequences were merged with a minimum overlap of 10 base pairs (bp) and zero mismatches. Chimeras were removed using the “consensus” method of the removeBimeraDenovo() function. Sequences were subjected to a final trimming to include only sequences between 245 and 258 bp. Taxonomic assignment was performed using the Silva v.138 database (Quast et al., 2013). Amplicon sequence variants (ASVs) with taxonomic assignments that were non-bacterial, unassigned at the kingdom level, chloroplasts, or mitochondria were removed from the final dataset. Phylogenetic trees were built using the DECIPHER and PHANGORN packages in R (Wright, 2016; Schliep et al., 2017, 2019). The ASV table, sample metadata, taxonomic information, and phylogenetic tree were analyzed using Phyloseq for visualization and statistical testing (McMurdie & Holmes, 2013). To calculate diversity metrics and the identity of taxa assigned to the core microbiota, we used a rarefied abundance table iteratively rarefied 1,000 times (Schloss, 2024a, 2024b).

### Statistical analysis

After verifying normality of residual error and equal variance assumptions using Shapiro-Wilk and Levene’s tests, respectively, we used a linear mixed model in the *lmer* package (Kuznetsova, Brockhoff & Christensen, 2017) to test differences in bacterial alpha-diversity (Shannon index, Chao1, and species richness) in the bee bread for colonies before and after miticide treatment, using site as a random effect (Y ~ Treatment * Timepoint + (1|Site)). Although the assumption of normality in residual error was violated for the Shannon index model, qqnorm and histograms of the distribution of residual errors showed only a slight deviation from normality and thus we still used a linear mixed model. For all statistical testing, we report alpha = 0.05 for statistical significance and alpha = 0.1 for marginal significance (as per Muff et al., 2022). Beta-diversity was assessed using weighted and unweighted Unifrac distances *via* the adonis function of the vegan package through a PERMANOVA (PERmutational Multivariate ANalysis Of VAriance) (Y_dist_ ~ Treatment * Timepoint + strata = Site) (Oksanen et al., 2013). PERMANOVA was chosen because it is a distance-based method to test for associations between multivariate data (*e.g*., microbiota) and covariates. It is the permutation-based extension of the multivariate ANOVA to partition within-group and between-group distances and is often used in the microbiome field to analyze beta-diversity patterns. PERMANOVA tests the null hypothesis that the centroids of groups are equal. To account for variation within each sampling location, we set the strata argument equal to ‘Site’ to restrict permutations within each apiary. Next, to quantify the effect of miticide treatment on the bee bread bacterial communities, we subset our dataset to include only those samples collected after application and used ‘Site’ in the models to block permutations (*i.e*., strata = Site). We used NMDS to visualize the beta-diversity results. Differences in the abundances of acidophilic bacteria across the three different miticide treatments were assessed by comparing the iteratively rarefied abundances of taxa assigned to the order Lactobacillales or family Acetobacteraceae. We used a Kruskal-Wallis test to determine the statistical significance of the effect of miticide treatment on taxon abundance, as this has been shown to possess appropriate statistical power when using rarefied counts (Yang & Chen, 2022). When significant, pairwise Wilcox tests were used to determine pairwise differences in taxon abundance among miticide treatments. All statistical analyses were conducted in the stats package in R version 4.2.2 (R Core Team, 2016).

### Core taxa assignments

A core set of bacterial taxa in bee bread was assigned by summarizing the rarefied taxon table at the genus level using the phyloseq package (McMurdie & Holmes, 2013). Next, we assigned core membership using the ‘taxa_core’ function in the phylosmith package (Smith, 2019). We defined core taxa based on the following criteria: (*i*) presence in 100% of sampling locations, (*ii*) presence in at least 50% of samples within each sampling location, and (*iii*) a relative abundance greater than 0.01% within each sample (Custer et al., 2023). Patterns of beta-diversity in the core were assessed using the same statistical and visualization approaches as for the entire dataset.

### Data accessibility

Raw sequence data was deposited in the NCBI Sequence Read Archive (SRA) under the bioproject accession number PRJNA940115. All codes used for sequence data processing and analyses are available at https://github.com/gcuster1991/Lawrence_et_al_2025_PeerJ (to be made public at the time of publication).

## Results

### Summary of sequence data

A total of 76.9% of total raw reads were retained following pre-processing with an average sequence depth of 8,020 reads per sample. ASV clustering using DADA2 resulted in a total of 1,337 ASVs, and the final dataset consisted of 941 ASVs after iterative rarefaction set to 1,100 reads per sample. This rarefaction threshold sufficiently covered richness, as shown in the rarefaction curves (Fig. S1). Bacterial taxa in the bee bread consisted of members from 18 unique phyla (Figs. S2 and S3). These taxa mostly included members within the phyla Proteobacteria (67.5% ± 24.0), Firmicutes (28.8% ± 25.1), Actinobacteriota (1.49% ± 1.4), and Bacteroidota (1.55% ± 2.3), representing a total of 364, 180, 128, and 125 ASVs, respectively. At the order level, we report 89 unique orders, with taxa mostly affiliated with Lactobacillales (26.4% ± 25.9), Cytophagales (0.4% ± 0.06), Rhizobiales (2.9% ± 2.2), Pseudomonadales (27.5% ± 14.4), and Burkholderiales (2.5% ± 1.9), each representing >50 ASVs.

### Bee bread bacterial alpha diversity

**Figure 2:**
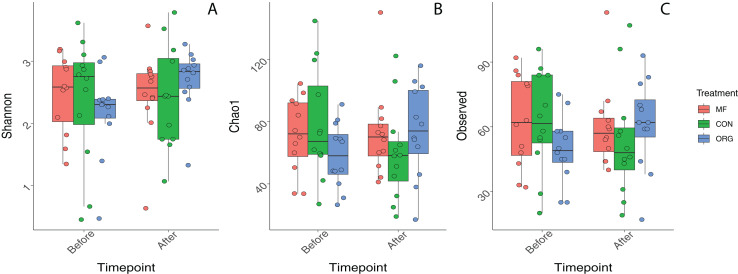
Boxplots depicting differences in bacterial alpha diversity before and after miticide treatments. Alpha diversity was calculated using (A) Shannon index, (B) Chao index, and (C) species richness. Colonies under miticide-free (pink), conventional (green), and organic (blue) management.

**Figure 3:**
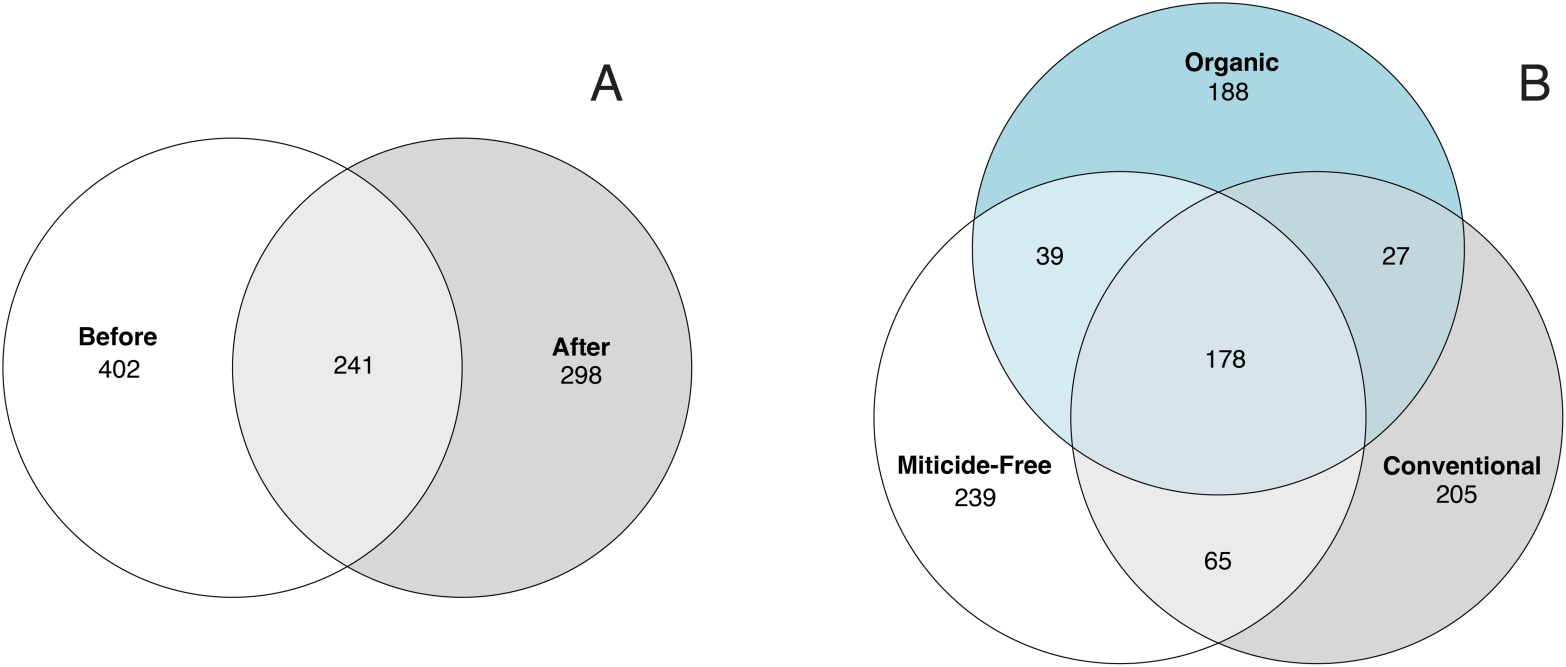
Venn diagram showing the number of unique and shared taxa. (A) Samples before and after miticide treatment, and (B) samples from different miticide treatments: control (miticide-free), conventional, and organic.

Variation in bacterial alpha diversity of bee bread was not predicted by treatment, timepoint, or their interactions, except for the interaction of sampling location and sampling timepoint that was a significant predictor of Chao1 index (F_2,34_ = 3.310, *p*-value = 0.043; Table S3; Fig. 2). No clear trends in the directionality of the interactions between miticide treatments, or sampling timepoints were observed in alpha-diversity metrics (Fig. 2C). Bee bread samples before and after treatments shared 25% of the bacterial diversity (Fig. 3A), while bee bread samples from colonies managed under different systems shared 18% of the taxa (Fig. 3B).

### Beta diversity of the bee bread microbiota

Timepoint explained 3.2% and 4% of the variation in bacterial β-diversity in bee bread for weighted and unweighted Unifrac distances, respectively (PERMANOVA weighted F_2,34_ = 2.266, *p*-value = 0.44; PERMANOVA unweighted F_2,34_ = 2.915, *p*-value < 0.001; Table S4). The significant effect of timepoint on bacterial community composition was consistent for unweighted distances regardless of the miticide effect (Table S5). We did not find statistically significant pairwise differences among miticide treatments when both timepoints were considered (Fig. S4). However, we found a marginally significant effect of treatment on the post-treatment samples for the unweighted Unifrac distance explaining 5.6% of the variation (PERMANOVA unweighted F_2,34_ = 0.981, *p*-value = 0.052; Table S6; Fig. S4), but not for weighted Unifrac (PERMANOVA weighted F_2,34_ = 0.416, *p*-value = 0.662; Table S6). The effect of miticide treatment on the community composition of the bee bread microbiota was driven by changes associated with the application of organic miticides (PERMANOVA MF *vs* ORG unweighted F_1,22_ = 1.255, *p*-value = 0.029; Table S7).

### Responses of acidophilic bacterial taxa to miticide treatment

To characterize the effect of organic and synthetic miticide treatments on acidophilic bacteria, we examined the abundances of taxa within the family Acetobacteraceae and the order Lactobacillales, at the second timepoint. We found a significant difference in the relative abundances of OTUs 122 (Acetobacteraceae; *Bombella* spp.) after miticide treatments ( ${\chi^2}$ > 6.125, df = 2, *p*-value = 0.004). The OTU 122 showed the highest relative abundance in colonies managed under the miticide-free and conventional systems, with the lowest relative abundance in the organic management system (Fig. S5; MF *vs* CON: *p*-value = 0.30, MF *vs* ORG: *p*-value < 0.01, CON *vs* ORG: *p*-value < 0.05).

### Defining the core bee bread microbiota

**Figure 4:**
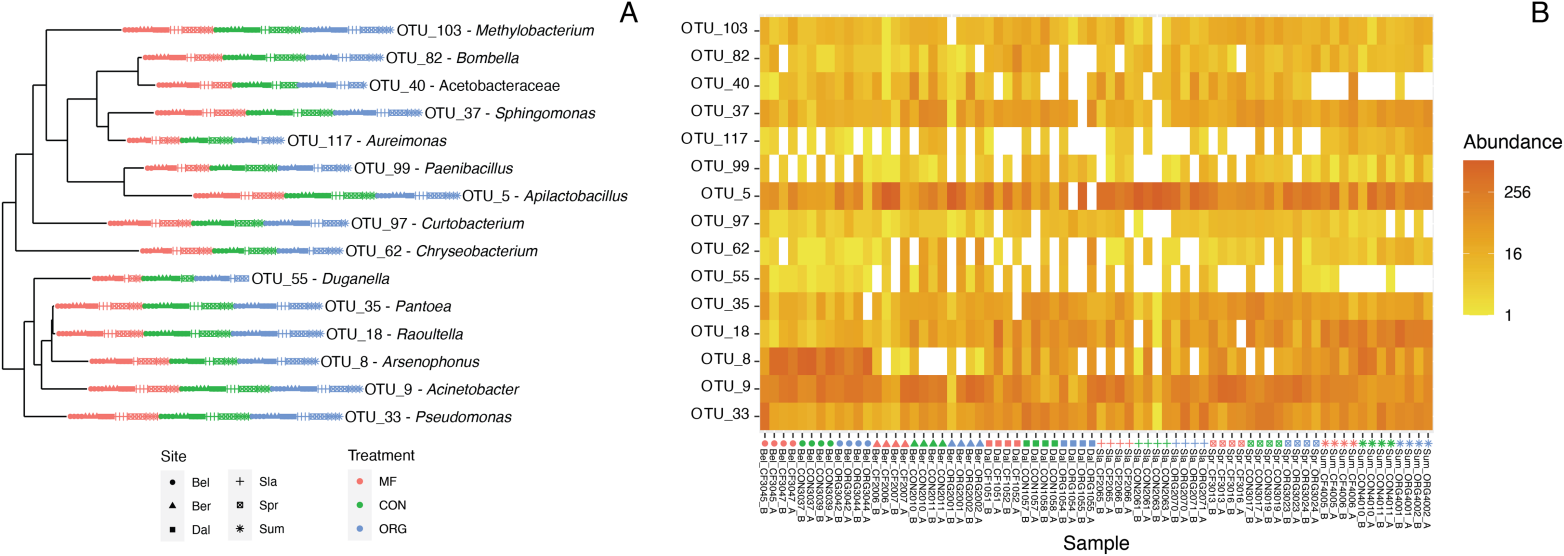
Characterization of the core bee bread microbiota. (A) Phylogenetic tree showing the genetic distance between the 15 taxa identified as part of the bee microbiome. (B) Heatmap depicting the relative abundance of the 15 core taxa across all 36 samples included in this study. Colors indicate miticide treatments (miticide-free (pink), conventional (green), and organic (blue)), and symbols indicate samples from different sites.

**Figure 5:**
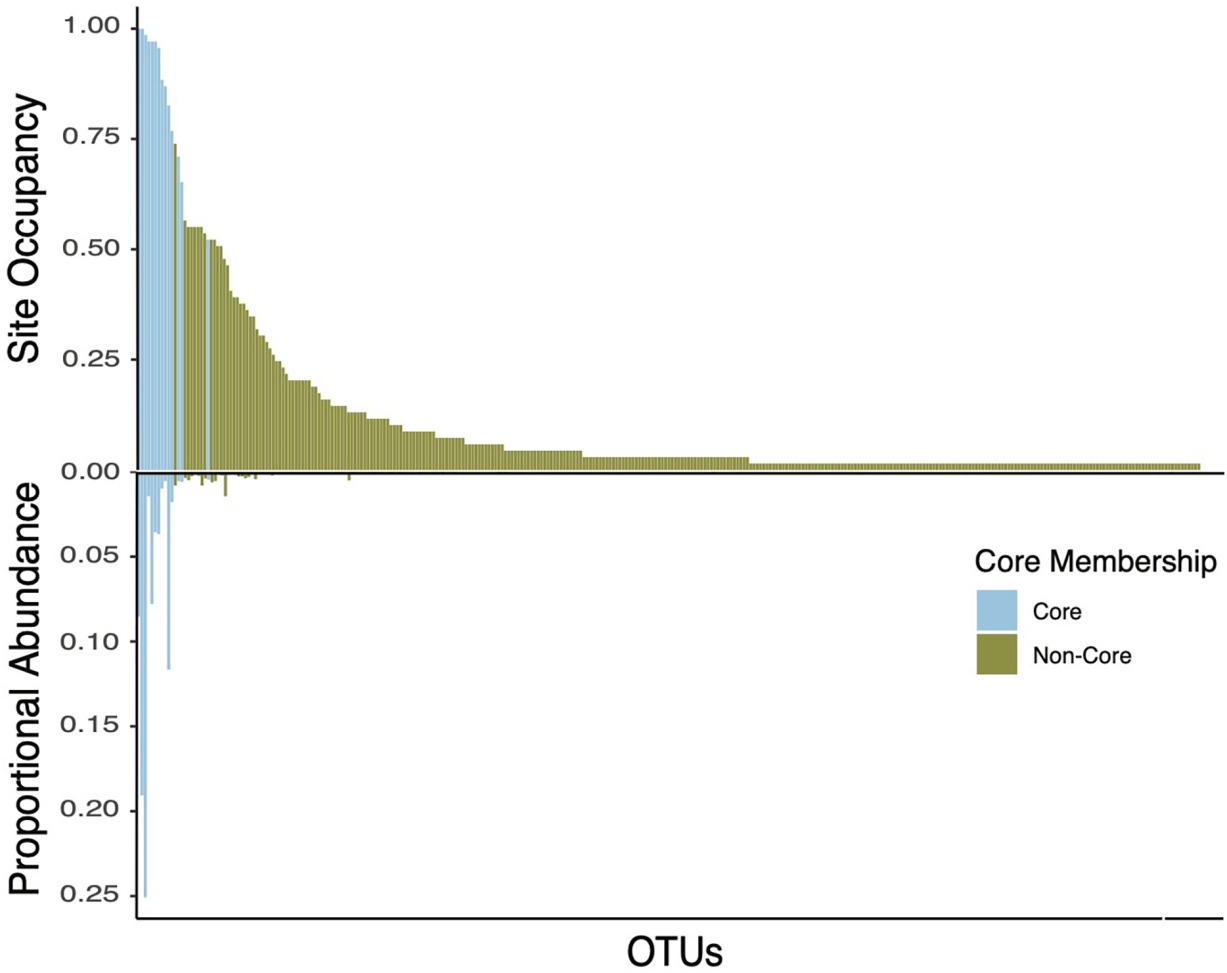
Double bar plot displaying the abundance and occupancy distribution of core and non-core bacterial taxa in bee bread. Bars above the black line at 0 indicate the taxon occupancy across sites (*e.g*., the proportion of sites at which each taxon was present). Bars below the black 0 line indicate the proportional abundance of total reads. Bars are colored by core inclusion status, with blue bars denoting core taxa (total of 15) and green bars denoting non-core taxa (total of 333).

The core taxa in bee bread of sampled colonies included 15 genera that met our criteria for thresholds in abundance and occupancy (Table 1; Fig. 4). While representing only 4.6% of the total genera in our dataset, the assigned core taxa accounted for 88.5% of the total reads (Fig. 5). The only predictor of the composition of the core microbiota was timepoint for the unweighted distances (PERMANOVA unweighted F_5,34_ = 6.919, *p*-value < 0.01; Table S8); however, this variable was only marginally significant based on weighted Unifrac (PERMANOVA weighted F_5,34_ =1.859, *p*-value = 0.070; Table S8). We did not find any significant effect of miticide applications on the beta-diversity of the core taxa of the bee bread microbiota (Table S9).

**Table 1:** Taxonomic assignments of the 14 bacterial taxa identified as core in honey bee bread based on the Silva database and NCBI classifications of 100% similarity of representative sequence^1^. Mean percent abundance (% Abun) and frequency (Freq) are also reported. Previous reports of these bacteria in bee bread and other honey bee microbiome studies are also referenced.

OTUID	Phylum	Class	Order	Family	Genus	NCBI 100% Hit	% Abun	Freq	References
5	Firmicutes	Bacilli	Lactobacillales	Lactobacillaceae	*Apilactobacillus*	*Apilactobacillus kunkeei*	23.92	0.97	Bee bread (Donkersley et al., 2018; Gorrochategui-Ortega et al., 2022; Santorelli et al., 2023)
8	Proteobacteria	γ Proteobacteria	Enterobacterales	Morganellaceae	*Arsenophonus*	*Arsenophonus apicola*	11.54	0.83	Bee bread (Donkersley et al., 2018; Gorrochategui-Ortega et al., 2022; Santorelli et al., 2023)
9	Proteobacteria	γ Proteobacteria	Pseudomonadales	Moraxellaceae	*Acinetobacter*	*Acinetobacter apis*	18.21	1.00	Bee bread (Donkersley et al., 2018)
18	Proteobacteria	γ Proteobacteria	Enterobacterales	Enterobacteriaceae	*Raoultella*	*Rosenbergiella epipactidis*	8.02	0.97	Bee bread (Disayathanoowat et al., 2020)
33	Proteobacteria	γ Proteobacteria	Pseudomonadales	Pseudomonadaceae	*Pseudomonas*	*Pseudomonas oryzihabitans*	8.58	1.00	Bee bread (Donkersley et al., 2018)
35	Proteobacteria	γ Proteobacteria	Enterobacterales	Erwiniaceae	*Pantoea*	*Pantoea agglomerans*	3.81	0.97	Bee bread (Disayathanoowat et al., 2020; Santorelli et al., 2023)
37	Proteobacteria	α Proteobacteria	Sphingomonadales	Sphingomonadaceae	*Sphingomonas*	*Sphingomonas paucimobilis*	3.63	0.97	Bee bread (Gorrochategui-Ortega et al., 2022)
40	Proteobacteria	α Proteobacteria	Acetobacterales	Acetobacteraceae	*Neokomagataea*	*Neokomagataea tanensis*	1.70	0.76	Bee bread (Santorelli et al., 2023)
55	Proteobacteria	γ Proteobacteria	Burkholderiales	Oxalobacteraceae	*Duganella*		0.57	0.57	
62	Bacteroidota	Bacteroidia	Flabobacteriales	Weekselllaceae	*Chryseobacterium*		0.77	0.74	
82	Proteobacteria	α Proteobacteria	Acetobacterales	Acetobacteraceae	*Bombella*		0.99	0.88	Bee bread (Gorrochategui-Ortega et al., 2022)
97	Actinobacteriota	Actinobacteria	Micrococcales	Microbacteriaceae	*Curtobacterium*	*Curtobacterium flaccumfaciens*	0.54	0.88	Hive entrance (Gorrochategui-Ortega et al., 2022)
99	Firmicutes	Bacilli	Paenibacillales	Paenibacillaceae	*Paenibacillus*	*Paenibacillus hordei*	0.51	0.75	Bee bread (Gorrochategui-Ortega et al., 2022)
103	Proteobacteria	α Proteobacteria	Rhizobiales	Beijerinckiaceae	*Methylobacterium*	*Methylobacterium radiotolerans*	1.39	0.97	Bee bread (Gorrochategui-Ortega et al., 2022)
117	Proteobacteria	α Proteobacteria	Rhizobiales	Rhizobiaceae	*Aureimonas*	*Aureimonas phyllosphaerae*	0.51	0.57	Hive entrance (Gorrochategui-Ortega et al., 2022)

**Note:**

1 Core microbiome is defined as taxa present in 100% of sampling locations, at least 50% of samples within each sampling location, and with a relative abundance of greater than 0.01% within each sample.

## Discussion

Here we report and characterize the diversity and community composition of the bee bread microbiota. We sampled bee bread from colonies located in apiaries placed in six sites, two time periods, and colonies treated with organic (formic acid) and synthetic (amitraz) miticides. Consistent with previous findings, bee bread samples varied significantly in the diversity and composition of the bacterial community (Smart et al., 2017; Santorelli et al., 2023). However, we found that 15 bacterial genera accounted for ~89% of all reads in the bee bread samples. These OTUs also tended to be the most frequent (present in over 50% of the hives and all sites) and included the genera *Apilactobacillus*, *Acinetobacter*, and *Arsenophonus*, with representative sequences assigned to *Apilactobacillus kunkeei*, *Acinetobacter apis*, and *Arsenophonus apicola*. Together, these three strains accounted for an average of 65% of all reads. The dominance of these bacterial genera is in line with culture-based results from previous studies of the bee bread microbiome (Anderson et al., 2014; Donkersley et al., 2018). It is known that lactic acid-producing bacteria are typical of fructose-rich environments, and are also commonly found in the gut of honey bees. In addition, these taxa have been shown to produce antifungal metabolites that promote host protection against pathogens (Iorizzo et al., 2020). The consistency of the composition of bee bread suggests honey bees exert strong control over the composition of bacterial taxa in the bee bread. This control may be expected to take one of two non-exclusive forms. First, honey bees might actively inoculate bee bread from their bodies (Corby-Harris, Maes & Anderson, 2014). And/or, second, like sourdough bread makers, honey bees may exert control over the conditions to which bee bread is subject and favor certain metabolic processes and the species that perform these specific functions (Reese et al., 2020).

Despite the significant sources of variation in sampling locations (sites), sampling periods, and miticide treatments across our samples, the bee bread microbiota of our samples was characterized by a consistent set of core taxa (Fig. 4). In contrast, the presence and relative abundance of non-core taxa were associated with factors such as timepoint and miticide applications. However, over 90% of the variation was not accounted for by either of these factors. Given that the primary component of bee bread is pollen, its microbiota is structured by the presence of site-specific plant-associated microbes (Anderson et al., 2013; Donkersley et al., 2018; Yang & Chen, 2022). In fact, pollen provides a unique microhabitat for bacteria, and there can be a high degree of species specificity as different textures on surfaces of the pollen serve as areas of colonization and growth for microbes (Ambika Manirajan et al., 2016). Studies of the microbiota of pollen provisions of honey bees and other bee species have reported a range of associations between pollen and microbial diversity of food provisions in nests. For example, in wood nesting bees in the genus *Ceratina*, the fungal communities associated with pollen varied geographically in association with plant diversity, but not for bacteria (McFrederick & Rehan, 2016, 2019). Because honey bees are highly polylectic species with one of the broadest dietary ranges among bees (Cane & Sipes, 2006), their bee bread microbiota are strongly structured by the variation in local pollen availability (Donkersley et al., 2018), and this is likely an important source of variation in the samples included in our study. Importantly, there is still a need to investigate the direct association between local floral availability and the microbiome associated with the foods stored in honey bee colonies. Despite the variability induced by local floral availability, the presence of a core set of taxa indicates that the environmental filters of bee bread may select for a consistent set of dominant taxa regardless of local conditions.

The effect of miticide application on bee bread bacterial communities was found to be marginally significant, even though we expected that organic acids (formic acid) would cause significant impacts on the nest environment and potentially favor acid-tolerant bacterial taxa. The relative abundances of core taxa were not affected by miticide treatment. Instead, miticide applications had only a modest effect on the relative abundance of non-core taxa. The only taxon that showed a significant decrease after the application of organic miticides was OTU 122 assigned to *Bombella* spp. Bacteria in this genus are acetic-acid organisms commonly found in the midgut of honey bee queens, as well as nectar and royal jelly (Tarpy, Mattila & Newton, 2015; Yun et al., 2017). Given our focus on bacterial communities, we cannot preclude the possibility that miticides can exert impacts on fungal taxa in bee bread, which also have key functions in the system (Anderson & Mott, 2023). Indeed, previous studies have shown that fungicide contamination reduces the number of fungal isolates from bee bread and that this reduction in fungal diversity facilitates the growth of pathogenic fungi in the system (Yoder et al., 2013). While the functional impact of the observed miticide-induced changes is unknown, minor changes in the number and abundance of rare bacteria may impact long-term pollen preservation in the colony (Anderson et al., 2013). At present, there is disagreement in the literature with regard to the functional role of the microbiota of the bee bread. On the one hand, a number of studies emphasize the relatively modest extent to which pollen appears to be transformed and metabolized in bee bread (Anderson et al., 2014). Conversely, other studies have shown that *Lactobacillus* spp. can metabolize components of the pollen in bee bread and facilitate the production of vitamins (Aylanc et al., 2021). Regardless, the dominance of lactic acid bacteria in the bee bread—particularly older bee bread—suggests that processes that involve the metabolism of sugars in anaerobic conditions and the reduction of the pH of the substrate are taking place and, likely make the bee bread inhospitable to some microbes (Di Cagno et al., 2019).

Given the increasing number of reports in the literature on simplified microbiome systems, it is possible to expand our discussion on bee bread microbiota in line with available studies on human-fermented foods (Dunn et al., 2021). In particular, studies of these systems suggest three possible models of how low-diversity core taxa are maintained. First, it is possible that bees inoculate the bee bread when nurse bees add secretions to the mixture of pollen and nectar before the food is stored in the cells (Corby-Harris, Maes & Anderson, 2014). This can perhaps more reliably explain the consistent core taxa, which include taxa common in the honey bee crop such as *A. kunkeei* (Olofsson & Vásquez, 2008; Anderson et al., 2013; Corby-Harris, Maes & Anderson, 2014; Yang & Chen, 2022). The inoculation(s) would likely rely on the effects of propagule pressure, wherein microbial taxa introduced at higher densities would more readily dominate the system. This model of inoculation is central to types of human fermentation that rely on back-slopping, as with yogurts and some types of beers and wines, or on the introduction of body-associated microbes, as with some types of sourdough bread, chicha, and kimchi (Marco et al., 2017). Another model is based on selective filtering events of environmental microbes from pollen and other sources that are subjected to the stringent extreme acidic conditions in the bee crop before being introduced into the bee bread (Anderson et al., 2013). Last, a third model is centered on the process of fermentation as a selective mechanism structuring the microbiota. Most likely, the outcome of the bee bread microbiota is determined by a dynamic interplay of these mechanisms operating over time.

While the microbiome of the honey bee gut has been extensively studied, the composition and function of the bee bread microbiota have received relatively less attention. We advocate such studies to be of critical importance to advance our understanding of microbial-mediated processes and how beekeeping management strategies impact the critically important food storage of the colony (Vásquez & Olofsson, 2009; Yoder et al., 2013; Anderson et al., 2013, 2014; Tarpy, Mattila & Newton, 2015; Donkersley et al., 2018; Disayathanoowat et al., 2020; Yang & Chen, 2022). Our study provides new information for beekeepers to consider as we identify changes in the bee bread bacterial communities that are correlated with the application of organic miticides. While the functional consequences of these changes remain to be determined, shifts in the bacterial communities of the bee bread microbiota can potentially impact the preservation processes that these bacteria provide to the stored pollen food in the colony (Anderson et al., 2014) or facilitate the growth of pathogenic species (Yoder et al., 2013).

## Conclusions

Our study reveals a consistent core microbiota within bee bread, dominated by key bacterial genera that likely play significant roles in the preservation of stored pollen. Despite variations in sampling locations, time periods, and miticide treatments, the dominance of genera such as *Apilactobacillus*, *Acinetobacter*, and *Arsenophonus* suggests that honey bees exert a strong influence over the microbial community in their food stores. Although miticide applications had no impact on core taxa, they affected some non-core taxa, indicating potential shifts in microbial dynamics that could influence the preservation and safety of bee bread. This underscores the need for further research into the functional roles of these communities, especially in relation to beekeeping practices and their implications for honey bee health and colony sustainability. Understanding the interactions between microbial diversity and environmental factors will be crucial for developing management strategies that support the resilience of bee populations.

## Supplemental Information

10.7717/peerj.20366/supp-1 Supplemental Information 1 Supplementary tables and figures.

## Additional Information and Declarations

### Field Study Permissions

The following information was supplied relating to field study approvals (*i.e*., approving body and any reference numbers):

All farm owners (6 farms) gave us verbal permission to place hives on their land for this experiment. The farmers were:

Dallas: Liza Rolland

Bernville: John Hartranft

Slatington: Glenn Rex

Belleville: Eli Peachey

Spring Mills: Christ Beiler

Summers: Milton Yoder

### Data Availability

The following information was supplied regarding data availability:

The raw sequence reads are available at NCBI: PRJNA940115.
