# Supplementary material for: The bee bread of honey bees is characterized by a core microbiota despite the application of miticide treatments and variation across space and time
Source: PeerJ. 2025 Nov 20;13:e20366. doi: 10.7717/peerj.20366 (PMC12640640; doi:10.7717/peerj.20366)
Supplement: Supplemental Information 1 [file peerj-13-20366-s001.docx]

**Supplemental Tables and Figures**

**
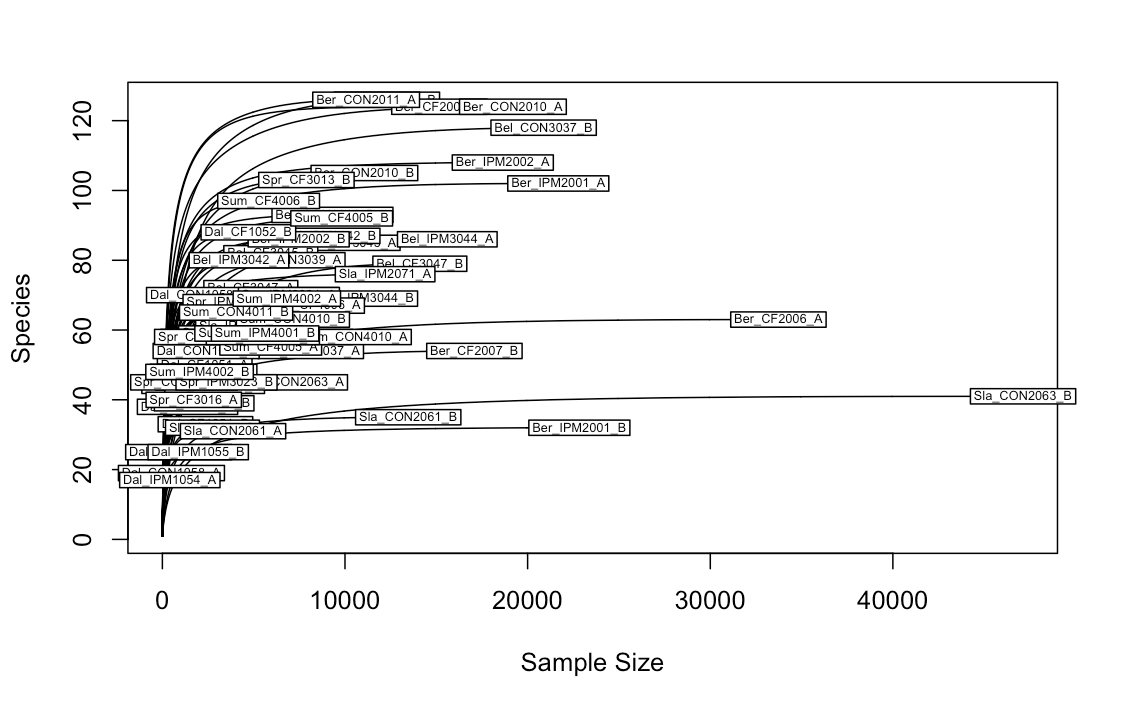
**

**Figure S1:** Rarefaction curve indicating that a threshold of 1,100 reads captured most of the bacterial richness in each sample.


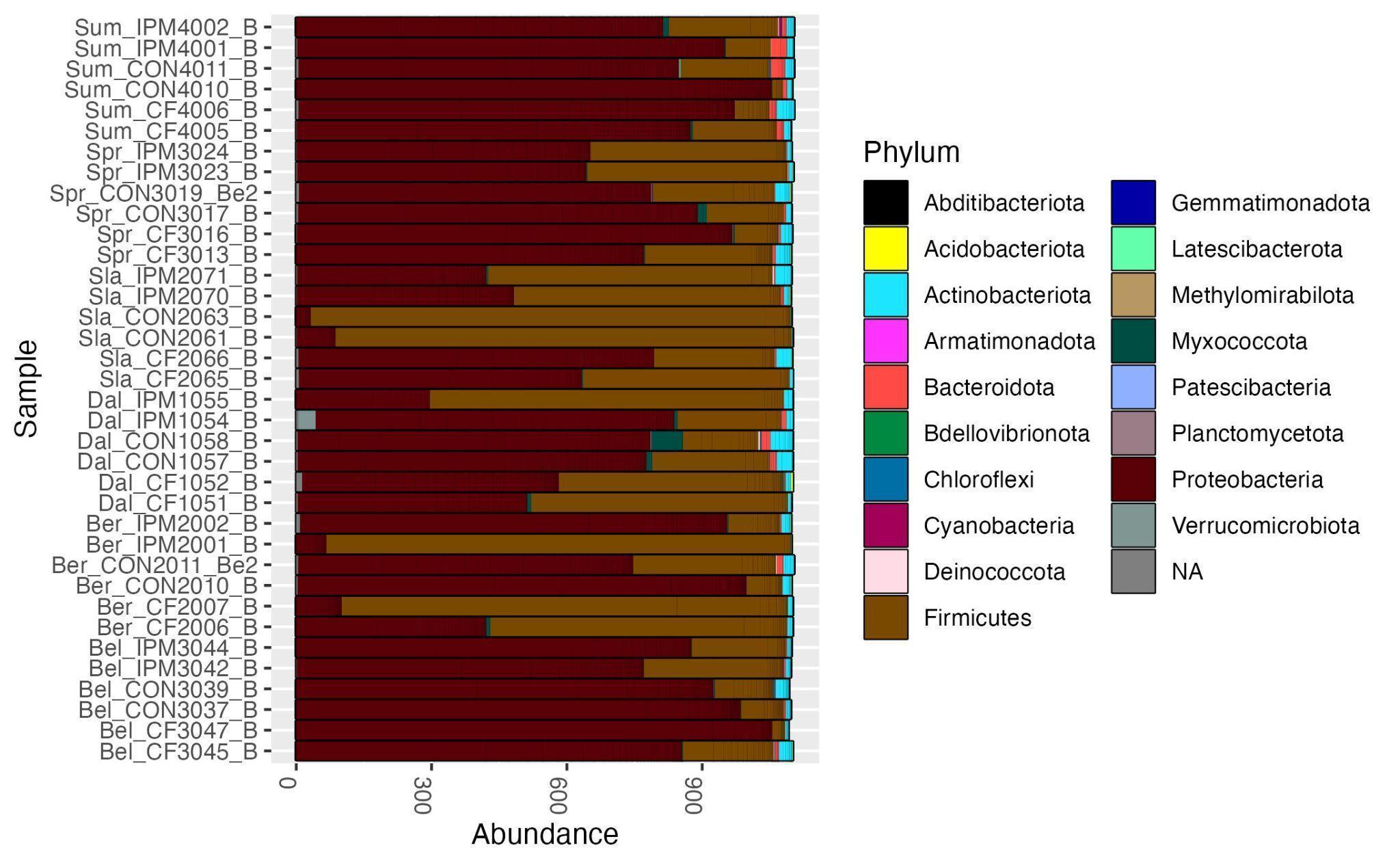


**Figure S2:** Phylum level relative abundance per colony for samples collected during timepoint 1 (before miticide treatment). Samples are grouped by sampling site.


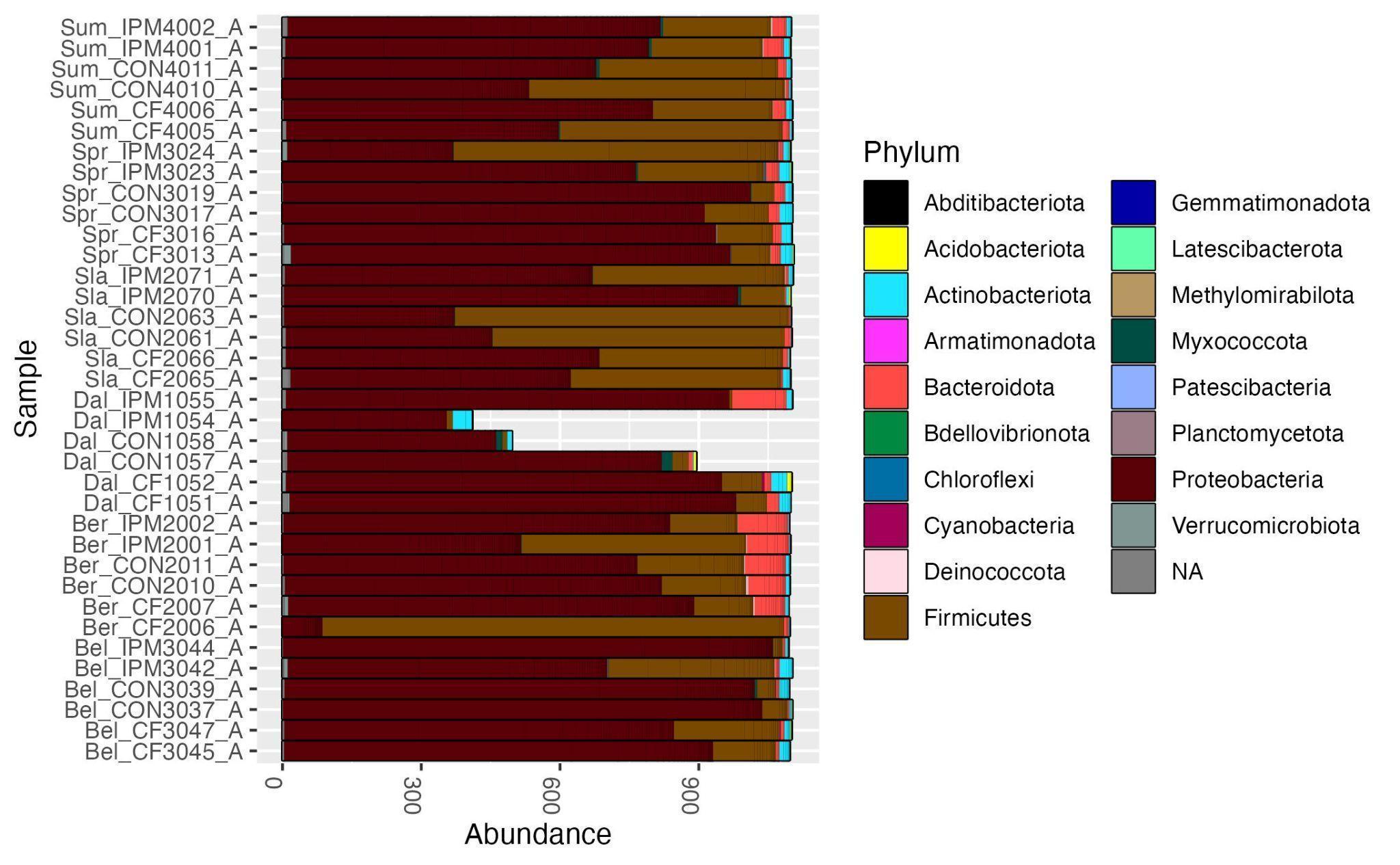


**Figure S3:** Phylum level relative abundance per colony for samples collected during timepoint 2 (after miticide treatment). Samples are grouped by sampling site.

**
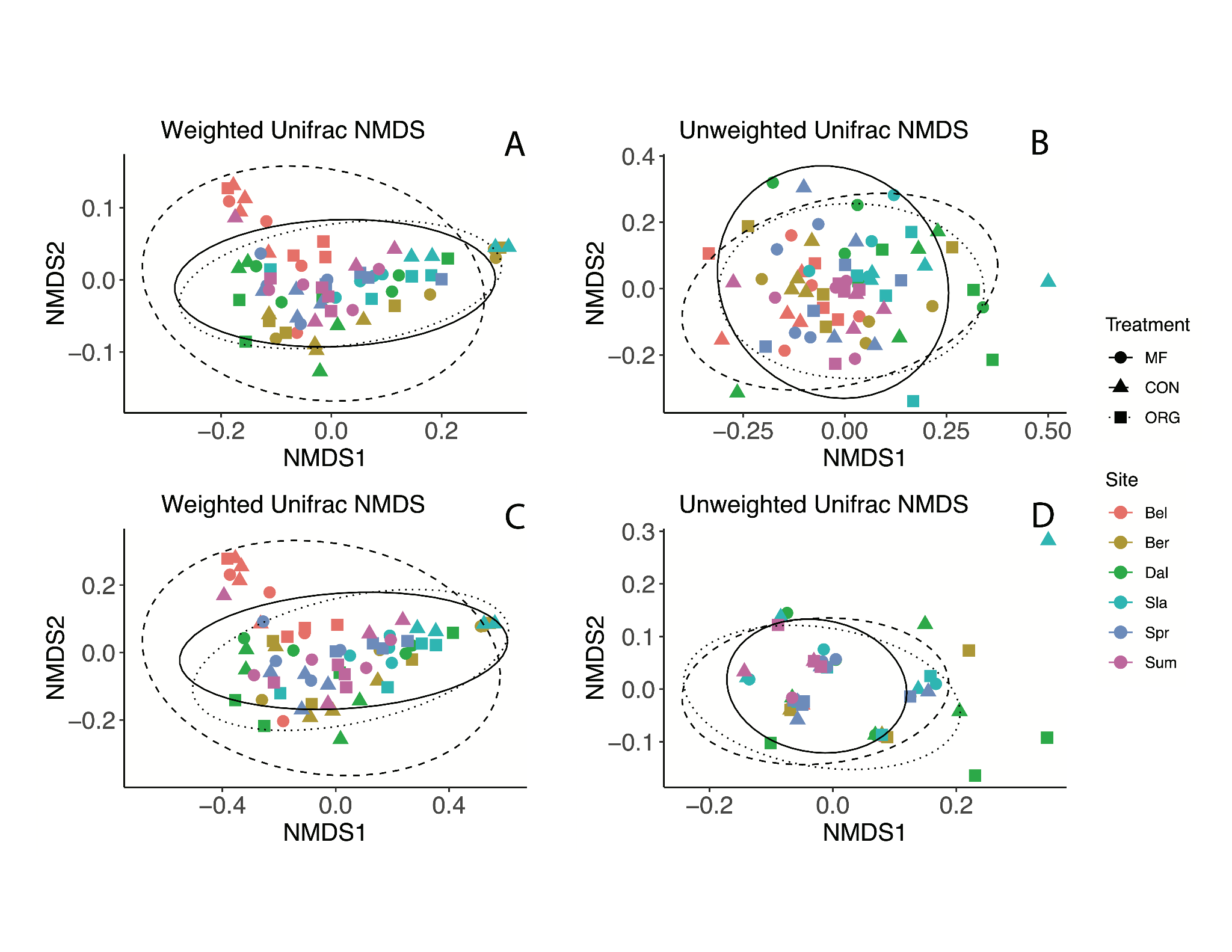
**

**Figure S4:** NMDS ordinations based on weighted and unweighted unifrac distances of bacterial communities in bee bread. Panels A and B include data from the entire dataset. Panels C and D represent samples post-treatment. Points are colored by sampling location, and shapes represent miticide treatment.


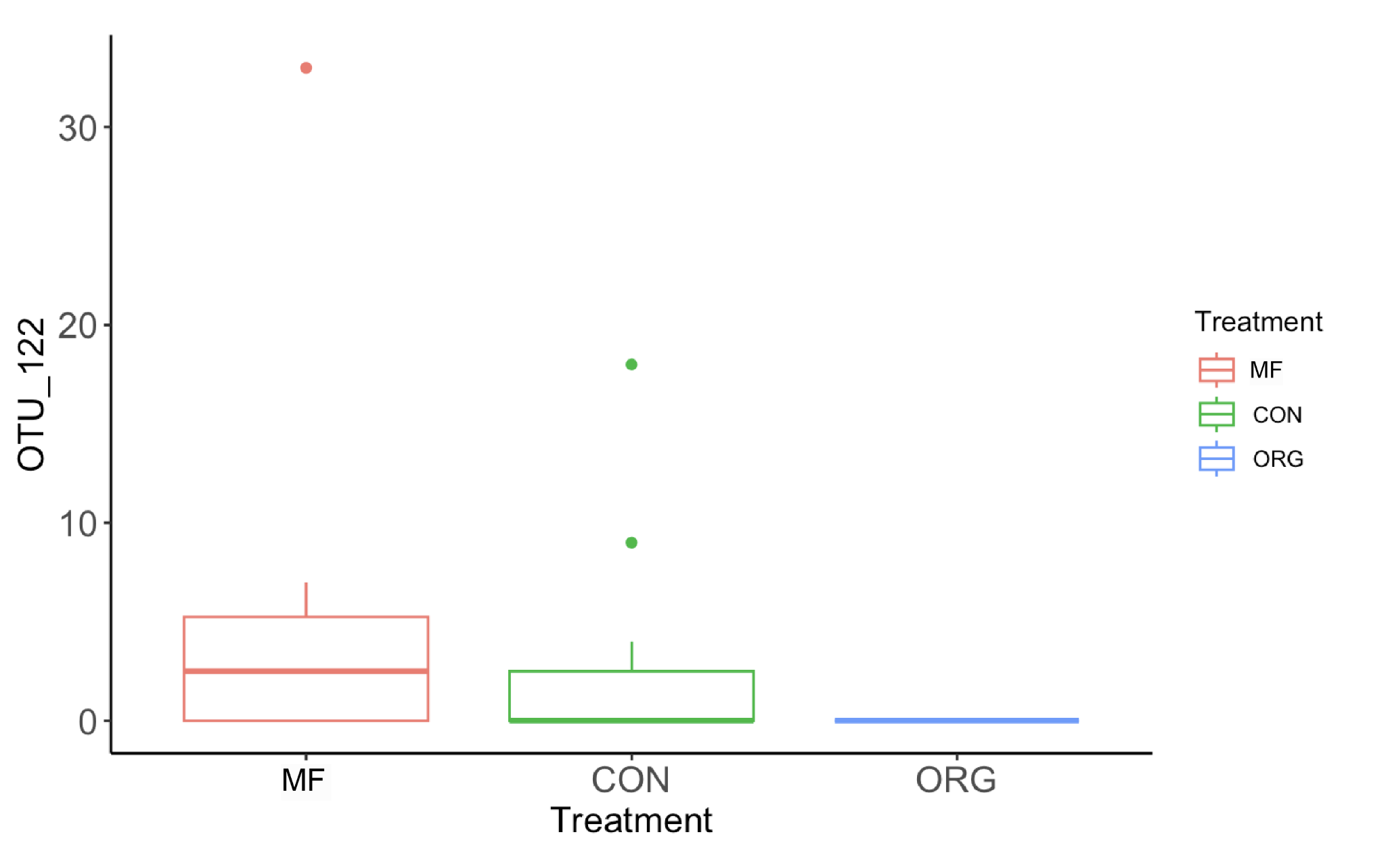


**Figure S5.** Boxplots show the number of copies of OUT 122 in colonies under the three different treatments miticide free (red), conventional (green) and organic (blue). Kruskal-Wallis rank sum test: chi-squash = 10.698; df = 2; p-value 0.0048). Wilcoxon rank sum test = MF vs CON: p-value = 0.30, MF vs ORG: p-value = 0.0032, CON vs. ORG: p-value = 0.033.

**Table S1:** Geographic coordinates of the six sites where the 36 honey bee colonies included in this study were placed. Sites included five farms in Pennsylvania and one in West Virginia (USA).

| **State** | **Location** | **Latitude** | **Longitude** |
| --- | --- | --- | --- |
| Pennsylvania | Belleville | 40.615 | -77.746 |
|  | Spring Mills | 40.857 | -77.620 |
|  | Slatington | 40.7303 | -75.634 |
|  | Bernville | 40.462 | -76.171 |
|  | Dallas | 41.267 | -75.986 |
| West Virginia | Summer County | 37.605 | -80.799 |

**Table S2:** Description of the three beekeeping management systems used in our experimental honey bee colonies.

|  | **Miticide-Free** | **Conventional** | **Organic** |
| --- | --- | --- | --- |
| Comb Foundation | 4.9 mm comb | 5.4 mm comb | 5.4 mm comb |
| Comb arrangement of brood chamber | 90% small-cell comb, 10% comb choice | 100% standard comb | 88.5% standard comb, 11.5% drone brood for removal |
| Bottom board | Solid | Screened | Solid |
| Queen Excluder | Absent | Present | Absent |
| Inner cover | Cotton duck cloth | Wintering cover | Wintering cover |
| Hive interior | Scratched for propolis attachment | Smooth | Smooth |
| Installation miticide treatment | None | Oxalic acid dribble 3 d after installation | Oxalic acid dribble 3 d after installation |
| Fall miticide treatment | None | Amitraz (brand name Apivar™) | Formic acid (brand name Formic Pro™) and drone brood removal |

**Table S3:** Results of linear mixed modeling estimating the effects of treatment, sampling time, and their interactions on bacterial alpha-diversity metrics in bee bread. Sampling location (‘site’) was used as a random effect. Alpha-diversity was quantified using Shannon diversity (H’), Chao1, and species richness. Numbers in bold indicate statistical significance at alpha= 0.05.

|  |  | **Shannon (H’)** | | **Chao1** | | **Richness** | |
| --- | --- | --- | --- | --- | --- | --- | --- |
| Variable | DF | F | P | F | P | F | P |
| Treatment | 2 | 0.059 | 0.942 | 0.351 | 0.705 | 0.697 | 0.505 |
| Timepoint | 1 | 1.227 | 0.272 | 0.043 | 0.835 | <0.01 | 0.994 |
| Treatment*Timepoint | 2 | 1.037 | 0.361 | 3.310 | **0.043** | 2.072 | 0.134 |

**Table S4:** Results of the PERMANOVA estimating the effect of treatment, sampling timepoint, and their interactions on community composition of the bee bread using iteratively rarefied abundances from 72 samples. The strata argument is set to sampling site. R-square, F-statistic, and p-values are reported for weighted and unweighted Unifrac distances. Numbers in bold indicate p-values significant at alpha= 0.05.

|  |  | **Weighted Unifrac** | | | **Unweighted Unifrac** | | |
| --- | --- | --- | --- | --- | --- | --- | --- |
| Variable | DF | R^2^ | F | P | R^2^ | F | P |
| Treatment | 2 | 0.016 | 0.587 | 0.494 | 0.026 | 0.9432 | 0.306 |
| Timepoint | 1 | 0.032 | 2.266 | **0.044** | 0.040 | 2.915 | **<0.001** |
| Treatment*Timepoint | 2 | 0.011 | 0.407 | 0.736 | 0.026 | 0.974 | 0.247 |

**Table S5:** Results of the pairwise PERMANOVA estimating the effect of treatment and sampling timepoint on pairwise differences in community composition of the bee bread using iteratively rarefied abundances from 72 samples. The strata argument is set to sampling location (‘site’). R-square, F-statistic, and p-values are reported for weighted and unweighted unifrac distances. Numbers in bold indicate p-values significant at alpha= 0.05, and italics indicate significance at alpha= 0.1.

|  |  | **Weighted Unifrac** | | | **Unweighted Unifrac** | | |
| --- | --- | --- | --- | --- | --- | --- | --- |
| Variable | DF | R^2^ | F | P | R^2^ | F | P |
| MF vs CON | | | | | | | |
| Treatment | 1 | 0.015 | 0.745 | 0.276 | 0.019 | 0.947 | 0.344 |
| Timepoint | 1 | 0.020 | 0.942 | 0.203 | 0.048 | 2.340 | **<0.001** |
| MF vs ORG | | | | | | | |
| Treatment | 1 | 0.005 | 0.237 | 0.839 | 0.021 | 1.041 | 0.186 |
| Timepoint | 1 | 0.047 | 2.221 | *0.074* | 0.049 | 2.391 | **<0.001** |
| CON vs ORG | | | | | | | |
| Treatment | 1 | 0.016 | 0.760 | 0.300 | 0.017 | 0.844 | 0.517 |
| Timepoint | 1 | 0.040 | 1.921 | *0.069* | 0.043 | 2.077 | **<0.001** |

**Table S6:** Results of the PERMANOVA estimating the effect of treatment on the composition of the bee bread microbiome in post-treated colonies using iteratively rarefied abundances from 36 colonies and strata set as location. The strata argument is set to sampling location (‘site’). R-square, F-statistic, and p-values are reported for weighted and unweighted Unifrac distances. Numbers in bold indicate p-values significant at alpha 0.05, and italics indicate significance at alpha= 0.1.

|  |  | **Weighted Unifrac** | | | **Unweighted Unifrac** | | |
| --- | --- | --- | --- | --- | --- | --- | --- |
| Variable | DF | R^2^ | F | P | R^2^ | F | P |
| Treatment | 2 | 0.024 | 0.416 | 0.662 | 0.056 | 0.981 | *0.052* |

**Table S7:** Results of the pairwise PERMANOVA estimating the effect of treatment on pairwise differences in the bee bread community composition using within-sample transformed abundances of post-treatment colonies. The strata argument is set to sampling location (‘site’). R-square, F-statistic, and p-values are reported for weighted and unweighted unifrac distances. Numbers in bold indicate p-values significant at alpha= 0.05, and italics indicate significance at alpha= 0.1.

|  |  | **Weighted Unifrac** | | | **Unweighted Unifrac** | | |
| --- | --- | --- | --- | --- | --- | --- | --- |
| Variable | DF | R^2^ | F | P | R^2^ | F | P |
| MF vs CON | | | | | | | |
| Treatment | 1 | 0.018 | 0.412 | 0.545 | 0.044 | 1.018 | 0.115 |
| MF vs ORG | | | | | | | |
| Treatment | 1 | 0.018 | 0.417 | 0.623 | 0.053 | 1.255 | **0.029** |
| CON vs ORG | | | | | | | |
| Treatment | 1 | 0.018 | 0.420 | 0.517 | 0.029 | 0.666 | 0.540 |

**Table S8:** Results of the PERMANOVA estimating the effect of treatment, sampling timepoint, and their interactions on the composition of the core bee bread using iteratively rarefied abundances from 72 colonies. The strata argument is set to sampling location (‘site’). Values for R-square, F-statistic, and p-values are reported for weighted and unweighted unifrac distances. Numbers in bold indicate p-values significant at alpha= 0.05, and italics indicate significance at alpha= 0.1.

|  |  | **Weighted Unifrac** | | | **Unweighted Unifrac** | | |
| --- | --- | --- | --- | --- | --- | --- | --- |
| Variable | DF | R^2^ | F | P | R^2^ | F | P |
| Treatment | 2 | 0.019 | 0.677 | 0.374 | 0.012 | 0.465 | 0.710 |
| Timepoint | 1 | 0.026 | 1.859 | *0.070* | 0.092 | 6.919 | **<0.01** |
| Treatment*Timepoint | 2 | 0.014 | 0.493 | 0.578 | 0.010 | 0.379 | 0.812 |

**Table S9:** Results of the PERMANOVA estimating the effect of treatment on composition of the core bee bread in pre- and post-treated colonies using rarefied abundances from 36 and 33 colonies, respectively. R-square, F-statistic, and p-values are reported for weighted and unweighted unifrac distances.

|  |  | **Weighted Unifrac** | | | **Unweighted Unifrac** | | |
| --- | --- | --- | --- | --- | --- | --- | --- |
| Variable | DF | R^2^ | F | P | R^2^ | F | P |
| Pre-treatment | | | | | | | |
| Treatment | 2 | 0.045 | 1.455 | 0.248 | 0.004 | 0.106 | 0.975 |
| Post-treatment | | | | | | | |
| Treatment | 2 | 0.016 | 0.472 | 0.757 | 0.040 | 1.340 | 0.3157 |
